# Comparison of four handheld point-of-care ultrasound devices by expert users

**DOI:** 10.1186/s13089-022-00274-6

**Published:** 2022-07-07

**Authors:** Minh-Phuong T. Le, Lara Voigt, Robert Nathanson, Anna M. Maw, Gordon Johnson, Ria Dancel, Benji Mathews, Alvaro Moreira, Harald Sauthoff, Christopher Gelabert, Linda M. Kurian, Jenna Dumovich, Kevin C. Proud, Jessica Solis-McCarthy, Carolina Candotti, Christopher Dayton, Alexander Arena, Brandon Boesch, Saul Flores, Mark T. Foster, Nicholas Villalobos, Tanping Wong, Gabriel Ortiz-Jaimes, Michael Mader, Craig Sisson, Nilam J. Soni

**Affiliations:** 1 Division of General & Hospital Medicine, Joe R. and Terry Lozano Long School of Medicine, University of Texas Health San Antonio, 7703 Floyd Curl Drive, MC 7885, San Antonio, TX 78229 USA; 2 Section of Hospital Medicine, South Texas Veterans Health Care System, San Antonio, TX USA; 3 Division of Hospital Medicine, University of Colorado, Aurora, CO USA; 4 Division of Hospital Medicine, Legacy Healthcare System, Portland, OR USA; 5 Division of Hospital Medicine, Department of Medicine, University of North Carolina, Chapel Hill, NC USA; 6 Division of Pediatric Hospital Medicine, Department of Pediatrics, University of North Carolina, Chapel Hill, NC USA; 7 Department of Hospital Medicine, HealthPartners Medical Group, Regions Hospital, St. Paul, MN USA; 8 Division of Neonatology, University of Texas Health San Antonio, Joe R. and Terry Lozano Long School of Medicine, San Antonio, TX USA; 9 Division of Pulmonary, Critical Care, and Sleep Medicine, Department of Medicine, New York University School of Medicine, New York, NY USA; 10 Department of Medicine, Division of Pulmonary and Critical Care, Veterans Affairs New York Harbor Healthcare System, New York, NY USA; 11 Department of Emergency Medicine, Division of Ultrasound, Joe R. and Terry Lozano Long School of Medicine, University of Texas Health San Antonio, San Antonio, TX USA; 12 Division of Hospital Medicine, Zucker School of Medicine at Hofstra Northwell, New Hyde Park, NY USA; 13 Division of Pulmonary Diseases & Critical Care Medicine, Joe R. and Terry Lozano Long School of Medicine, University of Texas Health San Antonio, San Antonio, TX USA; 14 Section of Pulmonary Medicine, South Texas Veterans Health Care System, San Antonio, TX USA; 15 Division of Hospital Medicine, University of California Davis, Sacramento, CA USA; 16 Division of Hospital Medicine, Alameda Health System-Highland Hospital, Oakland, CA USA; 17 Division of Critical Care and Cardiology, Texas Children’s Hospital, Baylor College of Medicine, Houston, TX USA; 18 Department of Pulmonology and Critical Care Medicine, San Antonio Military Medical Center, JBSA Fort Sam Houston, San Antonio, TX USA; 19 Division of Hospital Medicine, Weill Cornell Medicine, New York, NY USA; 20 Division of Pulmonary & Critical Care Medicine, Mayo Clinic, Rochester, MN USA; 21 Research and Development Service, South Texas Veterans Health Care System, San Antonio, TX USA

**Keywords:** Handheld ultrasound, Point-of-care ultrasound, POCUS, Portable ultrasound diagnostic imaging, Procedures

## Abstract

**Background:**

Point-of-care ultrasound (POCUS) is rapidly becoming ubiquitous across healthcare specialties. This is due to several factors including its portability, immediacy of results to guide clinical decision-making, and lack of radiation exposure to patients. The recent growth of handheld ultrasound devices has improved access to ultrasound for many clinicians. Few studies have directly compared different handheld ultrasound devices among themselves or to cart-based ultrasound machines. We conducted a prospective observational study comparing four common handheld ultrasound devices for ease of use, image quality, and overall satisfaction. Twenty-four POCUS experts utilized four handheld devices (Butterfly iQ+™ by Butterfly Network Inc., Kosmos™ by EchoNous, Vscan Air™ by General Electric, and Lumify™ by Philips Healthcare) to obtain three ultrasound views on the same standardized patients using high- and low-frequency probes.

**Results:**

Data were collected from 24 POCUS experts using all 4 handheld devices. No single ultrasound device was superior in all categories. For overall ease of use, the Vscan Air™ was rated highest, followed by the Lumify™. For overall image quality, Lumify™ was rated highest, followed by Kosmos™. The Lumify™ device was rated highest for overall satisfaction, while the Vscan Air™ was rated as the most likely to be purchased personally and carried in one’s coat pocket. The top 5 characteristics of handheld ultrasound devices rated as being “very important” were image quality, ease of use, portability, total costs, and availability of different probes.

**Conclusions:**

In a comparison of four common handheld ultrasound devices in the United States, no single handheld ultrasound device was perceived to have all desired characteristics. POCUS experts rated the Lumify™ highest for image quality and Vscan Air™ highest for ease of use. Overall satisfaction was highest with the Lumify™ device, while the most likely to be purchased as a pocket device was the Vscan Air™. Image quality was felt to be the most important characteristic in evaluating handheld ultrasound devices.

**Supplementary Information:**

The online version contains supplementary material available at 10.1186/s13089-022-00274-6.

## Introduction

Point-of-care ultrasound (POCUS), or use of bedside ultrasound by a clinician to answer a specific diagnostic question or guide performance of an invasive procedure, is becoming more common across healthcare specialties. Historically, POCUS has been performed using portable cart-based ultrasound machines that offer a wide range of modalities and consistently generate high-quality images, but access to portable ultrasound machines has been a top barrier to POCUS use [1–6]. In recent years, ultraportable handheld ultrasound devices have emerged improving access to POCUS technology at a fraction of the cost, especially in resource-limited settings [7].

Handheld ultrasound devices have demonstrated similar accuracy compared to cart-based ultrasound machines for multiple applications including bedside procedures, such as thoracentesis and epidural analgesia, and diagnostic evaluation of left ventricular function, female reproductive organs, abdominal pathologies (ascites, hydronephrosis, abdominal aortic aneurysm), musculoskeletal system, and lungs/pleura [8–16]. Discrepant findings between handheld ultrasound devices and cart-based ultrasound machines were not clinically significant in these studies [17, 18]. In contrast, few studies have performed head-to-head comparisons of different handheld ultrasound devices. Though a simple comparison of available modes, settings, and probes of different handheld devices can be performed relatively easily [19], POCUS users are seeking comparative data on the performance of handheld ultrasound devices to guide purchasing decisions.

We compared four commonly available handheld ultrasound devices in the United States for ease of use, image quality, and overall satisfaction with a multidisciplinary group of POCUS experts as operators. Our secondary objective was to identify the characteristics that POCUS experts consider to be most important when comparing different handheld ultrasound devices for use in clinical practice.

## Methods

### Subjects and setting

We conducted a prospective observational study in December of 2021 during a 2-day in-person POCUS continuing medical education course. Twenty-four expert POCUS faculty specializing in emergency, critical care, hospital, pediatrics, and pulmonary medicine acquired three standard POCUS views using four commercially available handheld ultrasound devices on the same three standardized patients with body mass index < 24. The University of Texas Health San Antonio Investigational Review Board deemed this educational study to be non-regulated research.

### Protocol

Four handheld ultrasound devices with both low- and high-frequency transducer capabilities were compared: Butterfly iQ + ™ (Butterfly Network, Inc.) probe connected by a USB-C cable to an Apple iPhone™ (iPhone 11 Pro Max™); Kosmos™ (EchoNous, Inc.) probe connected by a cord to a proprietary tablet as one unit; Lumify™ (Philips Healthcare) connected by a USB-C cable to an Apple iPad™ (8th generation), and Vscan Air™ (GE Healthcare) connected wirelessly to a Samsung Galaxy S7™ tablet.

Each standardized patient was assigned to one of three POCUS views: Focused Assessment with Sonography in Trauma (FAST) right upper quadrant view, transverse view of the neck with the internal jugular vein and carotid artery, or parasternal long-axis view of the heart. Using the four handheld devices, all 24 POCUS experts independently acquired the same view on the same model. For the parasternal long-axis view, experts were instructed to use the low-frequency probe with a cardiac preset and focus on the mitral valve, aortic valve, and endocardial lining. Color flow Doppler was then applied over the mitral and aortic valves. For the right upper quadrant FAST view, experts were instructed to use the low-frequency probe with an abdominal preset and focus on the liver, kidney, and diaphragm. For the transverse view of the neck, experts were instructed to use the high-frequency probe with a venous or vascular preset and focus on the internal jugular vein and common carotid artery.

### Data collection

Data were collected on ease of use, image quality, and overall satisfaction (Additional file 1). For ease of use, experts rated the physical characteristics, software navigation, maneuverability of the probe and tablet for imaging, and overall satisfaction. For image quality, experts rated the detail resolution, contrast resolution, penetration, clutter, and overall satisfaction. The overall ranking assessed satisfaction and recommendation for purchase. Ratings were made using standardized statements on a Likert scale of 1 (“strongly disagree” or “very dissatisfied”) to 5 (“strongly agree” or “very satisfied”). Qualitative feedback was collected in each category using free text. Experts completed the data collection form after scanning each model (< 72 h). All data were captured electronically using REDCap™ (Vanderbilt University, Nashville, TN, USA).

### Data analysis

Descriptive statistics were reported as frequencies with percentage for categorical variables and medians with interquartile range (IQR) for continuous data. Differences between medians were assessed by Kruskal–Wallis *H* test. Post hoc analysis was performed with Dunn’s test using a Bonferroni correction for multiple comparisons of groups. The rank analysis was performed via Friedman’s test followed by a post hoc Sign test for paired data. Spearman correlation coefficients were calculated to evaluate the correlation between experts’ prior experience with using each handheld device and ratings for ease-of-use, image quality, and overall satisfaction. A *p* value < 0.05 denoted statistical significance. All analyses were performed with R statistical software version 4.0.2.

Free text participant responses were analyzed using a qualitative deductive and inductive coding process aligned with a framework method approach [20]. The framework for deductive codes included advantages and disadvantages of all four handheld devices. We allowed for new codes that inductively arose from the data. Two investigators (AMM and JD) began the analysis by immersing in the data and developing the initial coding framework while coding free text responses of two surveys together. They then independently applied the coding framework to a subset of transcripts, reconvening multiple times to identify differences in coding, resolve them through discussion and refine the coding framework based on that discussion.

## Results

Twenty-four POCUS experts specializing in internal medicine/hospital medicine, emergency medicine, critical care medicine, pulmonary medicine, and pediatrics participated in this study. Most experts (71%) had completed a dedicated POCUS certification or fellowship, and most had been practicing medicine and using POCUS for > 5 years (Table 1).

**Table 1:** Characteristics of the point-of-care ultrasound expert users

Characteristic	All experts (%) *n* = 24
Specialty^a^	
Internal medicine/hospital medicine	13 (54)
Emergency medicine	6 (25)
Critical care medicine	6 (25)
Pulmonary medicine	4 (17)
Pediatrics	2 (8)
Female	8 (33)
United States region	
South (TX)	13 (54)
East (NY, NC)	5 (21)
West (CA, OR, CO)	4 (17)
Midwest (MN)	2 (8)
Completed ultrasound fellowship or certificate program^b^	
Yes	17 (71)
Clinical experience in practice	
0–5 years	5 (21)
6–10 years	8 (33)
> 10 years	11 (46)
Experience using point-of-care ultrasound	
0–5 years	4 (17)
6–10 years	14 (58)
> 10 years	6 (25)
Applications routinely used^c^	
Procedural guidance	22 (92)
Heart	23 (96)
Lungs/pleura	24 (100)
Abdomen	22 (92)
Vascular	22 (92)
Skin/soft tissues	18 (75)

^a^Six experts have two different specialties

^b^Includes completion of a dedicated ultrasound fellowship, the Chest or Society of Hospital Medicine’s POCUS certificate programs, or testamur status of the Advanced Critical Care Echocardiography or General Echocardiography by the National Board of Echocardiography

^c^Experts were allowed to select more than one application and each application represents a percentage of 24 experts

Ratings of the handheld ultrasound devices’ ease of use and image quality are displayed in Table 2. For ease of use, handheld devices were rated on physical characteristics, software navigability, maneuverability, and overall ease of use. Vscan Air™ was rated highest for physical characteristics and maneuverability, while Butterfly iQ + ™ was rated highest for software navigability. Overall ease of use was highest with the Vscan Air™.

**Table 2:** Ratings of handheld ultrasound devices per point-of-care ultrasound experts

Variable	Rating per experts (*n* = 24) [mean (sd)]				p-value
	Butterfly iQ+™	Kosmos™	Lumify™	Vscan Air™	
Ease of use					
Physical characteristics	3.75 (0.9)	3.54 (0.8)	4.17 (0.6)	4.42 (0.6)	< 0.001
Software	4.38 (0.7)	3.79 (0.8)	4.17 (0.7)	4.25 (0.9)	0.059
Maneuverability	4.38 (0.7)	3.63 (1.0)	4.00 (0.9)	4.58 (0.6)	0.002
Overall satisfaction (ease of use)	3.96 (1.0)	3.63 (0.7)	4.08 (0.7)	4.25 (0.8)	0.028
Image quality					
Detail resolution	3.17 (1.0)	4.46 (0.5)	4.67 (0.5)	4.33 (0.8)	< 0.001
Contrast resolution	3.13 (1.0)	4.33 (0.6)	4.58 (0.5)	4.21 (0.8)	< 0.001
Penetration	3.00 (1.0)	4.46 (0.7)	4.42 (0.8)	4.13 (0.8)	< 0.001
Clutter	2.67 (1.0)	4.29 (0.6)	4.42 (0.5)	3.79 (0.8)	< 0.001
Overall satisfaction (image quality)	2.92 (1.2)	4.25 (0.7)	4.46 (0.5)	4.08 (1.0)	< 0.001

Ratings of 1–5 based on a Likert scale of agreement (5) or disagreement (1)

*p*-values from Kruskal–Wallis rank sum test; < 0.05 indicates at least one device is statistically different from another device

**Fig. 1:**
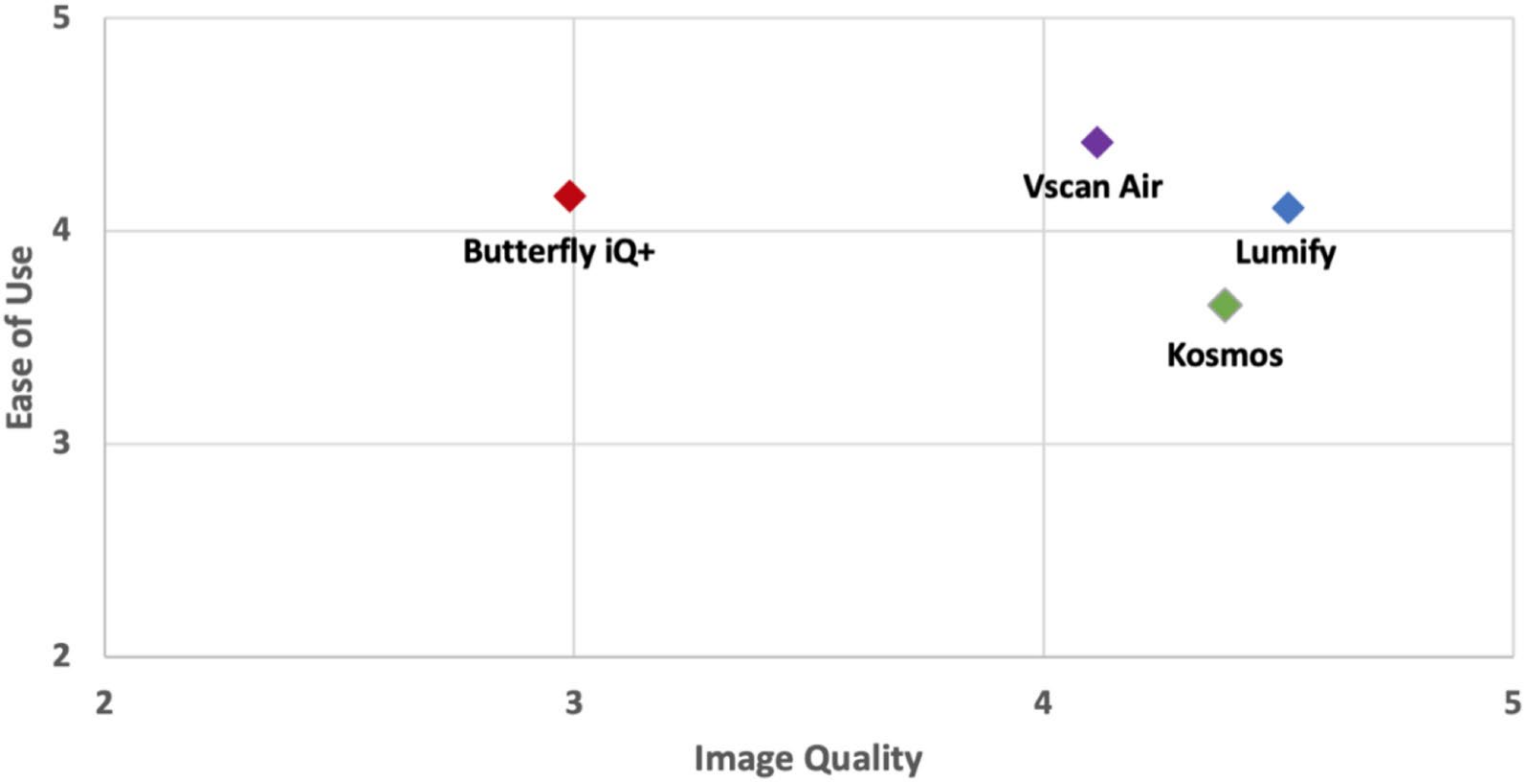
Mean ratings of handheld ultrasound devices by ease of use and image quality

**Fig. 2:**
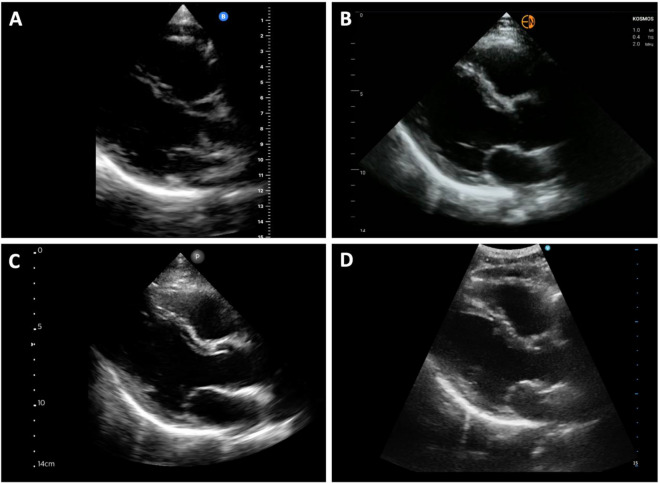
Parasternal long-axis view by four handheld ultrasound devices. Parasternal long-axis views acquired from the same standardized patient in early systole with the mitral valve closed and aortic valve open are shown from the **A** Butterfly IQ+™ (Butterfly Network, Inc.), **B** Kosmos™ (Echonous, Inc.), **C** Lumify™ (Phillips Healthcare), and **D** Vscan Air™ (General Electric)

For image quality, the handheld devices were rated on detail resolution, contrast resolution, penetration, clutter, and overall satisfaction with image quality. Lumify™ was rated highest for detail resolution, contrast resolution, and clutter, while Kosmos™ was rated highest for penetration. Overall image quality was highest for Lumify™. The mean ratings for ease of use and image quality are displayed in Fig. 1. Examples of the parasternal long-axis view captured in early systole using the four handheld ultrasound devices are shown in Fig. 2.

**Fig. 3:**
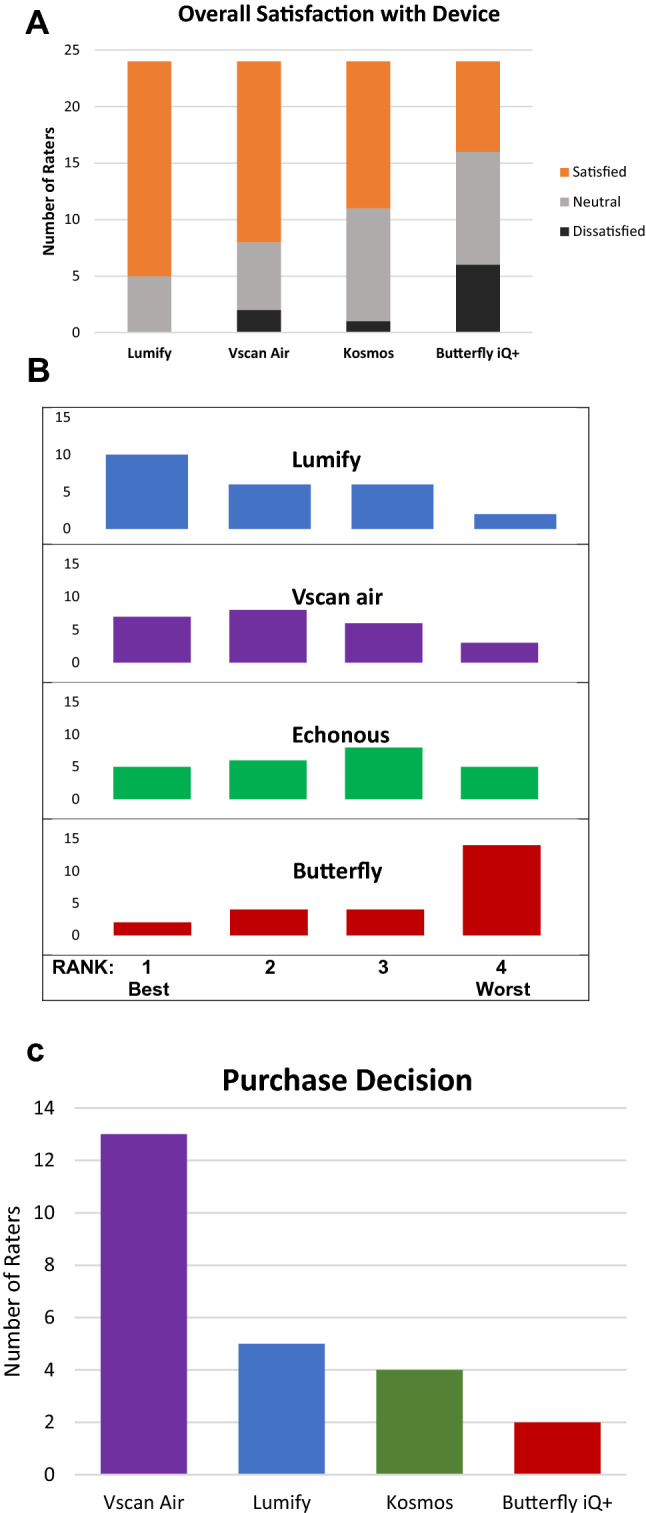
**A** Overall satisfaction with handheld ultrasound devices per POCUS Experts. Experts’ responses were defined as Satisfied for "I like this device and would definitely use it in patient care", Neutral for “I don’t have strong feeling for or against this device. I might use it in patient care,” and Dissatisfied for “I would not use this device even if it was given to me for free.” **B** Overall Ranking of Handheld Ultrasound Devices by POCUS Experts. **C** Recommendation for Purchase of a Personal Handheld Ultrasound Device by POCUS Experts

In the final section, experts were asked about their overall satisfaction with each handheld device. The Lumify™ and Vscan Air™ received the highest number of “satisfied” responses (Fig. 3A). Furthermore, experts ranked the four devices in order from 1 (“best”) to 4 (“worst”) and the Lumify™ and Vscan Air™ received the highest rankings (Fig. 3B). When experts were asked if they would purchase or recommend purchase of a specific handheld, a “yes” response was received most often for Lumify™ followed by Vscan Air™. However, when asked which device experts would “buy today to carry in their coat pocket,” the Vscan Air™ was most frequently selected (Fig. 3C).

Experts were asked about the most important characteristics of handheld devices (Additional file 3: Table S2). The top five most important characteristics were image quality, ease of use, portability, total costs, and availability of different probes. We evaluated for potential bias due to prior exposure to a device and found no association between experts’ experience levels and their ratings for a device’s ease-of-use or image quality (Additional file 2: Table S1). There also was no association between experience and overall satisfaction for three of the devices. However, a weak negative association was identified for the Vscan Air™, indicating that experts with some experience with the device were less satisfied with it than experts with no experience. None of the experts had extensive experience with the Vscan Air™.

From the qualitative data, two main themes emerged. First, there is not yet a perfect handheld device. Experts perceived all four devices as having notable advantages and disadvantages. Experts valued high image quality, the convenience of having only one probe, intuitive user interfaces, and advanced features, like Doppler ultrasound and artificial intelligence (AI). Ultimately, no single device was perceived as having all desired qualities or features. Second, adequate image quality was considered the most important aspect of a handheld device because it determined the user’s ability to make a clinical decision. Multiple experts commented that poor image quality limited clinical decisions and for this reason devices with poor image quality were considered unusable by some experts. One expert stated, “The poor image quality is a deal breaker,” and another said, “Image quality is poor and would result in me getting a larger machine to confidently make a clinical decision which is double the work in my mind.” Some experts also noted poor image quality may negatively affect the ability for inexperienced POCUS users to attain competency. One expert remarked, “This (device) is being marketed to physicians with minimal training, and if you take a minimally trained physician and give them a device that is very poor quality, then you have a recipe for disaster”.

## Discussion

We directly compared four common handheld ultrasound devices and found Lumify™ was rated highest for overall image quality, while Vscan Air™ was rated highest for overall ease of use. The overall ranking, satisfaction, and recommendation for purchase was highest with Lumify™ followed by Vscan Air™, and Vscan Air™ was most often selected for use as a personal pocket device. No single handheld ultrasound device was perceived to have all desired characteristics. We explored the characteristics of handheld ultrasound devices that are considered most important from the perspective of expert POCUS users, and image quality was felt to be the most important.

Handheld ultrasound devices offer unique advantages, including greater portability and ease of disinfection [7, 15, 21, 22], but few studies have directly compared different handheld ultrasound devices. One study compared three different handheld devices with the same operator performing gynecological ultrasound exams [12]. In contrast, our study has important advantages and adds substantively to our understanding of handheld ultrasound devices. First, we had POCUS experts use the same four handheld ultrasound devices on the same standardized patients to control for potential patient, device, and operator variables that could confound results. Second, POCUS experts were asked to use a variety of presets using both high- and low-frequencies for common diagnostic and procedural views. Third, our study included 24 POCUS experts as operators from 5 different specialties. Thus, our data may be more generalizable across common POCUS applications and different specialties. Our study sought to quantitatively assess ease of use and image quality based on user experience and provide a global rating of overall satisfaction and recommendation for purchase. We believe our study better replicates the myriad of considerations that clinicians face when making a purchasing decision.

Ultimately, no single handheld ultrasound device was rated highest in all categories. A lack of consensus of a single handheld ultrasound device being superior to others was similar to the findings by Toscano et al. in which investigators sought one suitable handheld device to perform gynecologic ultrasound exams in a resource-poor setting and ultimately selected the Lumify™ based on its ease of use, battery-life, portability, cost, and ease with depth and gain adjustments [12]. Even though POCUS experts rated Lumify™ highest for “overall satisfaction” in our study, more experts selected Vscan Air™ as the device they would buy “today as a personal device to carry in my coat pocket”.

Our study sought to identify specific characteristics of handheld ultrasound devices that expert POCUS users considered important. Quantitative and qualitative data demonstrated image quality to be the most important characteristic. Comments from experts reflected that image quality is the most important characteristic because poor quality images require a repeat evaluation with a cart-based ultrasound machine, negating the benefits of having a handheld. After image quality, the most important characteristics of handheld devices were ease of use, portability, total costs, and availability of different probe types. Perhaps once image quality is adequate for clinical decision-making, these secondary characteristics, such as ease of use and software options, become more of a deciding factor in selecting a handheld ultrasound device. Some of the characteristics, such as portability and number probes, are inherently related. A deeper understanding of the characteristics that POCUS users consider important, as well as the variables that affect purchasing decisions, warrant further investigation and may guide manufacturers in product development.

We recognize our study has limitations. First, POCUS experts could not be blinded to the different handheld devices and completed their scanning of the 3 standardized patients in the same room. Despite the large size of the room, we could not prevent experts from sharing their thoughts about the devices which could have biased their ratings. Further, our POCUS experts were not provided with dedicated training on the different handheld ultrasound devices, and lack of training may have limited the experts’ ability to navigate the software and optimize image quality. Third, bias from prior experience with some of the devices may have been a factor in the experts’ evaluations; however, we did not find an association between experts’ prior experience and the overall satisfaction ratings for three devices (Additional file 2: Table S1). Although there was a weak association detected between the experts’ experience level and overall satisfaction for the Vscan Air™, the association was negative, indicating that familiarity with the device did not inflate the ratings from experts. Fourth, experts rated handheld devices against one another, but we did not assess whether each handheld device met a minimal acceptable standard for ease of use and image quality. Additionally, we did not evaluate all potential diagnostic applications, including lungs, first trimester pregnancy, bladder, and lower extremity deep venous thrombosis, and we did not evaluate the handheld devices for procedural applications. Finally, POCUS users will need to consider several factors that were not explored in our study, including total costs of device over its lifespan; compatibility with preferred smart phone or tablet; probe characteristics (ergonomics, overheating, wired versus wireless connectivity); battery life; quality assurance and image archiving options; and desire for remote teleguidance or artificial intelligence software. Thus, handheld ultrasound purchasing decisions are complex, involving individual user preferences and device features, as well as external factors, such as institutional device restrictions and purchasing contracts, which were not addressed in our study.

For clinicians, the implications of our findings are threefold. First, though Vscan Air™ was rated highest for overall ease-of-use and Lumify™ for overall image quality, a perfect handheld device that combines all desired features does not currently exist. Second, when rating the importance of sixteen characteristics of handheld ultrasound devices, image quality was the only characteristic rated by all twenty-four experts as being “very important,” and new users should consider giving image quality priority when evaluating devices. Third, we focused our comparison of handheld ultrasound devices using 2-dimensional imaging alone. However, handheld ultrasound technology is advancing rapidly with new features, including artificial intelligence to guide image acquisition and interpretation, image sharing capabilities for remote teaching and real-time image interpretation by off-site experts, and robotics to facilitate probe placement for diagnostic imaging and invasive procedures [16, 23]. Future studies will be needed to compare the advantages and disadvantages of these advanced technologies in handheld ultrasound devices.

## Conclusion

As more handheld ultrasound devices become available to clinicians, understanding the advantages and disadvantages of different devices is imperative. In our comparison of four common handheld ultrasound devices, we demonstrated that overall image quality was best with Lumify™, while ease of use was best with Vscan Air™. Though most POCUS experts recommended Lumify™ for purchase, Vscan Air™ was most often selected as a personal pocket device. Thus, no single handheld ultrasound device was perceived to be superior in all categories. Future comparative studies of handheld ultrasound devices shall consider advanced technologies, such as artificial intelligence and tele-ultrasound, and their impact on training and patient outcomes.

## Supplementary Information

**Additional file 1. **Comparison of handheld point-of-care ultrasound devices.

**Additional file 2: Table S1. **Individual expert’s experience with devices compared to ratings for overall satisfaction, image quality, and ease-of-use.

**Additional file 3: Table S2. **Importance of characteristics for a handheld ultrasound device.

## Data Availability

The datasets used and/or analyzed during the current study are available from the corresponding author on reasonable request.

## Abbreviations

POCUS: Point-of-care ultrasound
FAST: Focused assessment with sonography in trauma
