# Supplementary material for: Comparison of four handheld point-of-care ultrasound devices by expert users
Source: Ultrasound J. 2022 Jul 7;14:27. doi: 10.1186/s13089-022-00274-6 (PMC9263020; doi:10.1186/s13089-022-00274-6)
Supplement: Supplementary file 1 — Additional file 1. Comparison of handheld point-of-care ultrasound devices. [file 13089_2022_274_MOESM1_ESM.pdf]

Comparison of Handheld Point-of-care Ultrasound Devices

Goal: Gather quantitative and qualitative feedback from expert POCUS users on ease of use, image quality, and overall satisfaction of the most common handheld ultrasound devices available in the United States.

Aims:

Assess the ease of use of 4 common handheld ultrasound devices based on physical set-up while scanning, intuitiveness of software application, weight, and maneuverability with one hand. Assess image quality based on detail resolution (clarity of 2 small structures), contrast resolution (differentiate structures of different shades of gray), penetration (ability to clearly see deep structures), and clutter (suppression of acoustic clutter/noise). Evaluate overall satisfaction of 4 different handheld devices and explore determinants of an expert user's satisfaction.

Please press "Submit" to BEGIN or "Save & Return Later" to RETURN at a later time (You will be sent an email to a unique link that will save your information).

Demographics

First Name: __________________________________

Last Name: __________________________________

What is your primary specialty that you practice clinically?

- ◯ Hospital Medicine
- ◯ Emergency Medicine
- ◯ Critical Care Medicine
- ◯ Pulmonary/Critical Care Medicine
- ◯ Anesthesiology
- ◯ Primary Care
- ◯ Other

If Other specialty, please specify: __________________________________

Years in practice after completing your specialty training?

- ◯ 0
- ◯ 1
- ◯ 2
- ◯ 3
- ◯ 4
- ◯ 5
- ◯ 6
- ◯ 7
- ◯ 8
- ◯ 9
- ◯ 10
- ◯ 11
- ◯ 12
- ◯ 13
- ◯ 14
- ◯ 15
- ◯ 16
- ◯ 17
- ◯ 18
- ◯ 19
- ◯ 20
- ◯ 21
- ◯ 22
- ◯ 23
- ◯ 24
- ◯ 25
- ◯ 26
- ◯ 27
- ◯ 28
- ◯ 29
- ◯ 30
- ◯ 31
- ◯ 32
- ◯ 33
- ◯ 34
- ◯ 35
- ◯ 36
- ◯ 37
- ◯ 38
- ◯ 39
- ◯ 40
- ◯ 41
- ◯ 42
- ◯ 43
- ◯ 44
- ◯ 45
- ◯ 46
- ◯ 47
- ◯ 48
- ◯ 49
- ◯ 50
- ◯ 51
- ◯ 52
- ◯ 53
- ◯ 54
- ◯ 55
- ◯ 56
- ◯ 57
- ◯ 58
- ◯ 59
- ◯ 60
- ◯ 61
- ◯ 62
- ◯ 63
- ◯ 64
- ◯ 65
- ◯ 66
- ◯ 67
- ◯ 68

○ 69
○ 70
○ 71
○ 72
○ 73
○ 74
○ 75
○ 76
○ 77
○ 78
○ 79
○ 80
○ 81
○ 82
○ 83
○ 84
○ 85
○ 86
○ 87
○ 88
○ 89
○ 90
○ 91
○ 92
○ 93
○ 94
○ 95
○ 96
○ 97
○ 98
○ 99

Number of years using point-of-care ultrasound (POCUS)?

- ◯ 0
- ◯ 1
- ◯ 2
- ◯ 3
- ◯ 4
- ◯ 5
- ◯ 6
- ◯ 7
- ◯ 8
- ◯ 9
- ◯ 10
- ◯ 11
- ◯ 12
- ◯ 13
- ◯ 14
- ◯ 15
- ◯ 16
- ◯ 17
- ◯ 18
- ◯ 19
- ◯ 20
- ◯ 21
- ◯ 22
- ◯ 23
- ◯ 24
- ◯ 25
- ◯ 26
- ◯ 27
- ◯ 28
- ◯ 29
- ◯ 30
- ◯ 31
- ◯ 32
- ◯ 33
- ◯ 34
- ◯ 35
- ◯ 36
- ◯ 37
- ◯ 38
- ◯ 39
- ◯ 40
- ◯ 41
- ◯ 42
- ◯ 43
- ◯ 44
- ◯ 45
- ◯ 46
- ◯ 47
- ◯ 48
- ◯ 49
- ◯ 50
- ◯ 51
- ◯ 52
- ◯ 53
- ◯ 54
- ◯ 55
- ◯ 56
- ◯ 57
- ◯ 58
- ◯ 59
- ◯ 60
- ◯ 61
- ◯ 62
- ◯ 63
- ◯ 64
- ◯ 65
- ◯ 66
- ◯ 67
- ◯ 68

◯ 69
◯ 70
◯ 71
◯ 72
◯ 73
◯ 74
◯ 75
◯ 76
◯ 77
◯ 78
◯ 79
◯ 80
◯ 81
◯ 82
◯ 83
◯ 84
◯ 85
◯ 86
◯ 87
◯ 88
◯ 89
◯ 90
◯ 91
◯ 92
◯ 93
◯ 94
◯ 95
◯ 96
◯ 97
◯ 98
◯ 99

Which POCUS applications do you use routinely (select all that apply)?	☐ procedural guidance<br>☐ cardiac<br>☐ pulmonary<br>☐ abdomen<br>☐ vascular<br>☐ skin/soft tissues

---

Please press "Submit" after you have completed the survey. You will not be able to go back to a survey after you have pressed "Submit." You will be taken back to your queue of surveys to review the progress made and to select the next survey.

Please press "Save & Return Later" if you would like to save the information you have entered and return at a later time. You will be prompted to another screen to enter your email address. You will be sent an email to a unique link that will save your information.

Butterfly

Instructions:

Obtain these 3 views Parasternal long-axis view with color Doppler over the mitral valve Right upper quadrant FAST view (liver, kidney diaphragm visible) Internal jugular vein & common carotid transverse view with color Doppler over both vessels Answer questions below to rate the device on: Ease of use Image quality Overall satisfaction

Do you have prior experience using this device?

- ◯ None - "I've never used this device before."
- ◯ Some - "I've used this device occasionally in patient care or teaching workshops, but I am not proficient in using it."
- ◯ Extensive - "I use this device on a regular basis and am familiar with it."
- ◯ Other

If Other, please explain: __________________________________

EASE OF USE

Rate your level of agreement with each of statements.

	Strongly agree	Agree	Neutral	Disagree	Strongly disagree
The physical characteristics of this device made it easy to use (size, weight, hold in hand vs. use of stand).	◯	◯	◯	◯	◯
The software was easy to navigate (changing modes, depth, gain).	◯	◯	◯	◯	◯
The device is easy to maneuver with one hand on the probe and one hand on the tablet/processor.	◯	◯	◯	◯	◯

Rate your level of satisfaction with the following statement.

	Very satisfied	Somewhat satisfied	Neutral	Somewhat dissatisfied	Very dissatisfied)
My overall satisfaction with the ease of using this device was	◯	◯	◯	◯	◯

Comments:

IMAGE QUALITY

Rate your level of agreement with each of statements.

	Strongly agree	Agree	Neutral	Disagree	Strongly disagree
The detail resolution (clarity of 2 small structures) was good enough to answer common clinical questions.	◯	◯	◯	◯	◯
The contrast resolution (differentiate structures of different shades of gray) was good enough to answer common clinical questions.	◯	◯	◯	◯	◯
The penetration (ability to see deep structures well) was good.	◯	◯	◯	◯	◯
The clutter (acoustic clutter/noise) on the screen was minimal.	◯	◯	◯	◯	◯

Rate your level of satisfaction with the following statement.

	Very satisfied	Somewhat satisfied	Neutral	Somewhat dissatisfied	Very dissatisfied
My overall satisfaction with the image quality of this device was	◯	◯	◯	◯	◯

Comments:

OVERALL SATISFACTION

Which statement best expresses your overall satisfaction with this device for use in patient care?

◯ DISSATISFIED - "I would not use this device even if was given to me for free."
◯ NEUTRAL - "I don't have strong feeling for or against this device. I might use it in patient care."
◯ SATISFIED - "I like this device and would definitely use it in patient care."

Please describe the characteristics that made you feel satisfied, neutral, or dissatisfied with this device:

What are the main ADVANTAGES and DISADVANTAGES of this device currently?

Assuming a similar purchasing price to other devices on the market, would you personally buy or recommend this device for use in patient care?

◯ Yes
◯ No

Why or Why not?

Please press "Submit" after you have completed the survey. You will not be able to go back to a survey after you have pressed "Submit." You will be taken back to your queue of surveys to review the progress made and to select the next survey.

Please press "Save & Return Later" if you would like to save the information you have entered and return at a later time. You will be prompted to another screen to enter your email address. You will be sent an email to a unique link that will save your information.

Lumify (Philips)

Instructions:

Obtain these 3 views Parasternal long-axis view with color Doppler over the mitral valve Right upper quadrant FAST view (liver, kidney diaphragm visible) Internal jugular vein & common carotid transverse view with color Doppler over both vessels   Answer questions below to rate the device on: Ease of use Image quality Overall satisfaction

Do you have prior experience using this device?

○ None - "I've never used this device before."
○ Some - "I've used this device occasionally in patient care or teaching workshops, but I am not proficient in using it."
○ Extensive - "I use this device on a regular basis and am familiar with it."
○ Other

If Other, please explain: __________________________________

EASE OF USE

Rate your level of agreement with each of statements.

	Strongly agree	Agree	Neutral	Disagree	Strongly disagree
The physical characteristics of this device made it easy to use (size, weight, hold in hand vs. use of stand).	○	○	○	○	○
The software was easy to navigate (changing modes, depth, gain).	○	○	○	○	○
The device is easy to maneuver with one hand on the probe and one hand on the tablet/processor.	○	○	○	○	○

Rate your level of satisfaction with the following statement.

	Very satisfied	Somewhat satisfied	Neutral	Somewhat dissatisfied	Very dissatisfied)
My overall satisfaction with the ease of using this device was	○	○	○	○	○

Comments:

IMAGE QUALITY

Rate your level of agreement with each of statements.

	Strongly agree	Agree	Neutral	Disagree	Strongly disagree
The detail resolution (clarity of 2 small structures) was good enough to answer common clinical questions.	◯	◯	◯	◯	◯
The contrast resolution (differentiate structures of different shades of gray) was good enough to answer common clinical questions.	◯	◯	◯	◯	◯
The penetration (ability to see deep structures well) was good.	◯	◯	◯	◯	◯
The clutter (acoustic clutter/noise) on the screen was minimal.	◯	◯	◯	◯	◯

Rate your level of satisfaction with the following statement.

	Very satisfied	Somewhat satisfied	Neutral	Somewhat dissatisfied	Very dissatisfied
My overall satisfaction with the image quality of this device was	◯	◯	◯	◯	◯

Comments:

OVERALL SATISFACTION

Which statement best expresses your overall satisfaction with this device for use in patient care?

◯ DISSATISFIED - "I would not use this device even if was given to me for free."
◯ NEUTRAL - "I don't have strong feeling for or against this device. I might use it in patient care."
◯ SATISFIED - "I like this device and would definitely use it in patient care."

Please describe the characteristics that made you feel satisfied, neutral, or dissatisfied with this device:

What are the main ADVANTAGES and DISADVANTAGES of this device currently?

____

Assuming a similar purchasing price to other devices on the market, would you personally buy or recommend this device for use in patient care?

◯ Yes
◯ No

Why or Why not?

Please press "Submit" after you have completed the survey. You will not be able to go back to a survey after you have pressed "Submit." You will be taken back to your queue of surveys to review the progress made and to select the next survey.

Please press "Save & Return Later" if you would like to save the information you have entered and return at a later time. You will be prompted to another screen to enter your email address. You will be sent an email to a unique link that will save your information.

Kosmos (Echonous)

Instructions:

Obtain these 3 views Parasternal long-axis view with color Doppler over the mitral valve Right upper quadrant FAST view (liver, kidney diaphragm visible) Internal jugular vein & common carotid transverse view with color Doppler over both vessels   Answer questions below to rate the device on: Ease of use Image quality Overall satisfaction

Do you have prior experience using this device?

- ◯ None - "I've never used this device before."
- ◯ Some - "I've used this device occasionally in patient care or teaching workshops, but I am not proficient in using it."
- ◯ Extensive - "I use this device on a regular basis and am familiar with it."
- ◯ Other

If Other, please explain: ______________________

EASE OF USE

Rate your level of agreement with each of statements.

	Strongly agree	Agree	Neutral	Disagree	Strongly disagree
The physical characteristics of this device made it easy to use (size, weight, hold in hand vs. use of stand).	◯	◯	◯	◯	◯
The software was easy to navigate (changing modes, depth, gain).	◯	◯	◯	◯	◯
The device is easy to maneuver with one hand on the probe and one hand on the tablet/processor.	◯	◯	◯	◯	◯

Rate your level of satisfaction with the following statement.

	Very satisfied	Somewhat satisfied	Neutral	Somewhat dissatisfied	Very dissatisfied)
My overall satisfaction with the ease of using this device was	◯	◯	◯	◯	◯

Comments:

IMAGE QUALITY

Rate your level of agreement with each of statements.

	Strongly agree	Agree	Neutral	Disagree	Strongly disagree
The detail resolution (clarity of 2 small structures) was good enough to answer common clinical questions.	◯	◯	◯	◯	◯
The contrast resolution (differentiate structures of different shades of gray) was good enough to answer common clinical questions.	◯	◯	◯	◯	◯
The penetration (ability to see deep structures well) was good.	◯	◯	◯	◯	◯
The clutter (acoustic clutter/noise) on the screen was minimal.	◯	◯	◯	◯	◯

Rate your level of satisfaction with the following statement.

	Very satisfied	Somewhat satisfied	Neutral	Somewhat dissatisfied	Very dissatisfied
My overall satisfaction with the image quality of this device was	◯	◯	◯	◯	◯

Comments:

OVERALL SATISFACTION

Which statement best expresses your overall satisfaction with this device for use in patient care?

◯ DISSATISFIED - "I would not use this device even if was given to me for free."
◯ NEUTRAL - "I don't have strong feeling for or against this device. I might use it in patient care."
◯ SATISFIED - "I like this device and would definitely use it in patient care."

Please describe the characteristics that made you feel satisfied, neutral, or dissatisfied with this device:

What are the main ADVANTAGES and DISADVANTAGES of this device currently?

____

Assuming a similar purchasing price to other devices on the market, would you personally buy or recommend this device for use in patient care?

◯ Yes
◯ No

Why or Why not?

Please press "Submit" after you have completed the survey. You will not be able to go back to a survey after you have pressed "Submit." You will be taken back to your queue of surveys to review the progress made and to select the next survey.

Please press "Save & Return Later" if you would like to save the information you have entered and return at a later time. You will be prompted to another screen to enter your email address. You will be sent an email to a unique link that will save your information.

V-scan Air (GE)

Instructions:

Obtain these 3 views Parasternal long-axis view with color Doppler over the mitral valve Right upper quadrant FAST view (liver, kidney diaphragm visible) Internal jugular vein & common carotid transverse view with color Doppler over both vessels   Answer questions below to rate the device on: Ease of use Image quality Overall satisfaction

Do you have prior experience using this device?

- ◯ None - "I've never used this device before."
- ◯ Some - "I've used this device occasionally in patient care or teaching workshops, but I am not proficient in using it."
- ◯ Extensive - "I use this device on a regular basis and am familiar with it."
- ◯ Other

If Other, please explain: __________________________________

EASE OF USE

Rate your level of agreement with each of statements.

	Strongly agree	Agree	Neutral	Disagree	Strongly disagree
The physical characteristics of this device made it easy to use (size, weight, hold in hand vs. use of stand).	◯	◯	◯	◯	◯
The software was easy to navigate (changing modes, depth, gain).	◯	◯	◯	◯	◯
The device is easy to maneuver with one hand on the probe and one hand on the tablet/processor.	◯	◯	◯	◯	◯

Rate your level of satisfaction with the following statement.

	Very satisfied	Somewhat satisfied	Neutral	Somewhat dissatisfied	Very dissatisfied)
My overall satisfaction with the ease of using this device was	◯	◯	◯	◯	◯

Comments:

IMAGE QUALITY

Rate your level of agreement with each of statements.

	Strongly agree	Agree	Neutral	Disagree	Strongly disagree
The detail resolution (clarity of 2 small structures) was good enough to answer common clinical questions.	◯	◯	◯	◯	◯
The contrast resolution (differentiate structures of different shades of gray) was good enough to answer common clinical questions.	◯	◯	◯	◯	◯
The penetration (ability to see deep structures well) was good.	◯	◯	◯	◯	◯
The clutter (acoustic clutter/noise) on the screen was minimal.	◯	◯	◯	◯	◯

Rate your level of satisfaction with the following statement.

	Very satisfied	Somewhat satisfied	Neutral	Somewhat dissatisfied	Very dissatisfied
My overall satisfaction with the image quality of this device was	◯	◯	◯	◯	◯

Comments:

OVERALL SATISFACTION

Which statement best expresses your overall satisfaction with this device for use in patient care?

◯ DISSATISFIED - "I would not use this device even if was given to me for free."
◯ NEUTRAL - "I don't have strong feeling for or against this device. I might use it in patient care."
◯ SATISFIED - "I like this device and would definitely use it in patient care."

Please describe the characteristics that made you feel satisfied, neutral, or dissatisfied with this device:

What are the main ADVANTAGES and DISADVANTAGES of this device currently?

____

Assuming a similar purchasing price to other devices on the market, would you personally buy or recommend this device for use in patient care?

◯ Yes
◯ No

Why or Why not?

Please press "Submit" after you have completed the survey. You will not be able to go back to a survey after you have pressed "Submit." You will be taken back to your queue of surveys to review the progress made and to select the next survey.

Please press "Save & Return Later" if you would like to save the information you have entered and return at a later time. You will be prompted to another screen to enter your email address. You will be sent an email to a unique link that will save your information.

FINAL Survey

Based on your overall impression of the 4 handheld devices, rank them in order from best to worst (1=Best; 4=Worst).

	Butterfly	Lumify	Echonous	Vscan air
1st Choice (Best)	◯	◯	◯	◯
2nd Choice	◯	◯	◯	◯
3rd Choice	◯	◯	◯	◯
4th Choice (Worst)	◯	◯	◯	◯

Comments:

If I had to buy a handheld ultrasound device today as my personal device that I would carry in my coat pocket, I would purchase:

◯ Butterfly
◯ Lumify
◯ Echonous
◯ Vscan air

Why? Briefly explain your rationale.

When evaluating a handheld ultrasound device, rate the importance of the following characteristics.

	Very important	Somewhat important	Not important
Probe size	◯	◯	◯
Wireless vs. wired probe	◯	◯	◯
Availability of different probe types (linear, phased array, curvilinear)	◯	◯	◯
Portability	◯	◯	◯
Ease of use	◯	◯	◯
Battery life	◯	◯	◯
Manufacturer's warranty	◯	◯	◯
Customer service with manufacturer based on prior experience	◯	◯	◯

Total costs	○	○	○
Option to make a 1-time purchase vs. pay an ongoing fee to use it	○	○	○
Modes available in addition to B-mode<br>a. M-mode<br>b. Color Doppler<br>c. Spectral Doppler	○	○	○
Software packages available (presets, calculations)	○	○	○
Image quality	○	○	○
Reputation of manufacturer	○	○	○
Carrying method (case vs. pocket)	○	○	○
Ability to connect probe to any tablet/phone vs. probe + processor connected as 1 unit	○	○	○

Any other important characteristics not listed above? Please describe.

What are some of the unique advantages and disadvantages of each of the handheld devices that come to the top of your mind?

Advantages Disadvantages
Kosmos ______ ______
Vscan Air ______ ______
Butterfly ______ ______
Lumify ______ ______

Please press "Submit" after you have completed the survey. You will not be able to go back to a survey after you have pressed "Submit." You will be taken back to your queue of surveys to review the progress made and to select the next survey.

Please press "Save & Return Later" if you would like to save the information you have entered and return at a later time. You will be prompted to another screen to enter your email address. You will be sent an email to a unique link that will save your information.
