# Supplementary material for: Comparison of four handheld point-of-care ultrasound devices by expert users
Source: Ultrasound J. 2022 Jul 7;14:27. doi: 10.1186/s13089-022-00274-6 (PMC9263020; doi:10.1186/s13089-022-00274-6)
Supplement: Supplementary file 2 — Additional file 2: Table S1. Individual expert’s experience with devices compared to ratings for overall satisfaction, image quality, and ease-of-use. [file 13089_2022_274_MOESM2_ESM.pdf]

Table S1. Individual Expert's Experience with Devices Compared to Ratings for Overall Satisfaction, Image Quality, and Ease-of-Use

	Butterfly iQ+™				Lumify™				Kosmos™				Vscan Air™			
**Expert**	*Exp*	*Sat*	*Qual*	*Ease*	*Exp*	*Sat*	*Qual*	*Ease*	*Exp*	*Sat*	*Qual*	*Ease*	*Exp*	*Sat*	*Qual*	*Ease*
**1**	3	1	2.50	4.33	2	3	4.00	3.67	1	3	5.00	3.33	1	2	4.00	5.00
**2**	3	1	1.00	4.33	3	3	5.00	4.00	1	3	5.00	3.33	2	3	4.75	5.00
**3**	3	3	4.75	5.00	1	2	4.75	3.33	1	2	4.75	3.33	1	3	4.75	5.00
**4**	2	1	2.50	4.00	3	3	4.25	3.67	2	2	4.00	3.67	1	3	3.75	3.67
**5**	1	3	3.25	4.00	2	3	4.00	4.00	1	3	4.00	3.67	1	3	4.00	4.00
**6**	2	1	3.50	4.67	2	2	5.00	5.00	2	2	5.00	4.00	2	1	2.00	4.67
**7**	3	3	3.00	5.00	1	2	4.00	3.33	1	3	5.00	4.33	2	1	3.50	4.00
**8**	3	2	2.50	3.33	1	3	4.50	4.33	1	3	4.75	3.67	1	3	4.75	4.00
**9**	2	2	3.50	4.33	2	3	4.75	4.67	1	2	4.00	2.33	1	3	4.75	4.67
**10**	3	3	3.75	4.67	1	3	4.00	3.67	2	3	4.00	3.67	1	2	3.75	3.00
**11**	1	3	4.00	4.33	1	2	5.00	4.00	1	2	4.25	3.33	2	2	4.00	3.33
**12**	3	2	3.25	4.67	2	3	5.00	4.67	1	2	4.25	4.00	2	2	4.25	4.00
**13**	3	2	1.00	3.00	3	3	4.00	3.00	1	3	4.00	3.67	1	3	4.00	4.00
**14**	3	2	2.50	4.67	3	3	4.75	4.67	2	2	3.50	4.00	1	3	4.00	5.00
**15**	3	3	3.25	5.00	1	3	4.00	4.00	1	3	5.00	4.00	1	3	5.00	5.00

**16**	1	2	3.00	4.00	1	3	5.00	4.33	1	3	5.00	2.67	1	3	5.00	4.33
**17**	2	2	2.75	4.00	1	3	4.00	4.00	1	3	4.00	4.33	1	3	4.00	4.67
**18**	3	2	3.75	4.00	2	2	3.75	3.00	1	1	4.25	2.67	1	3	3.75	4.67
**19**	2	2	2.50	3.00	2	3	5.00	5.00	1	2	3.50	4.00	2	3	5.00	5.00
**20**	3	3	3.50	4.67	3	3	4.50	5.00	1	3	5.00	3.67	1	2	4.75	5.00
**21**	2	1	3.00	2.67	2	3	4.75	4.33	2	3	4.75	4.33	2	3	4.00	4.00
**22**	3	3	4.00	4.00	3	3	4.75	5.00	2	2	4.00	3.67	1	3	4.50	4.67
**23**	1	2	2.75	4.33	1	3	4.75	4.00	2	3	4.50	5.00	2	2	2.75	5.00
**24**	2	1	2.25	4.00	2	3	5.00	4.00	2	2	3.75	3.00	2	3	3.75	4.33
r_S		0.18	0.01	0.36		0.31	0.09	0.25		-0.20	-0.31	0.29		-0.40	-0.28	-0.06
**p-val**		0.40	0.96	0.09		0.14	0.67	0.23		0.36	0.14	0.17		0.05	0.19	0.77

***Exp**: Experience: 3=Extensive ("I use this device on a regular basis"), 2=Some ("I've used this device occasionally"), 1=None ("I've never used this device before").*

***Sat**: Overall Satisfaction: 3=Satisfied ("I would use it in patient care"), 2=Neutral ("I might use it in patient care"), 1=Dissatisfied ("I would not use").*

***Qual**: The average of 4 categories of Image Quality, each ranked on a scale of 1 to 5, with 5 indicating the most satisfaction.*

***Ease**: The average of 3 categories of Ease-of-Use, each ranked on a scale of 1 to 5, with 5 indicating the most satisfaction.*

*r_S is the Spearman correlation coefficient, and **p-val** is the p-value of the test of association based on the Spearman correlation coefficient.*
