# Supplementary material for: Comparison of four handheld point-of-care ultrasound devices by expert users
Source: Ultrasound J. 2022 Jul 7;14:27. doi: 10.1186/s13089-022-00274-6 (PMC9263020; doi:10.1186/s13089-022-00274-6)
Supplement: Supplementary file 3 — Additional file 3: Table S2. Importance of characteristics for a handheld ultrasound device. [file 13089_2022_274_MOESM3_ESM.pdf]

Table S2. Importance of Characteristics for a Handheld Ultrasound Device

Characteristic	Very Important	Somewhat Important	Not Important
**5 Most Important**			
Image Quality	24	0	0
Ease of Use	20	4	0
Portability	19	5	0
Total Costs	17	7	0
Availability of Different Probes	17	6	1
**Intermediate Importance**			
Battery Life	15	7	2
Probe Size	13	10	1
Availability of Additional Modes	11	13	0
Ability to Connect to Any Tablet/phone	10	11	3
Availability of Software Packages	9	13	2
Customer Service (prior experience)	8	15	1
**5 Least Important**			
Option for 1-time Purchase	9	12	3
Manufacturer's Warranty	6	16	2
Carrying Method	8	11	5
Reputation of Manufacturer	6	15	3
Wireless vs Wired	7	12	5
